# Postsurgical Refractory Gastroparesis: Response to Botulinum Toxin Therapy

**DOI:** 10.7759/cureus.6596

**Published:** 2020-01-08

**Authors:** Deepika Sarvepalli, Mamoon Ur Rashid, Waqas Ullah, Yousaf Zafar, Abu H Khan

**Affiliations:** 1 Internal Medicine, Guntur Medical College, Guntur, IND; 2 Internal Medicine, AdventHealth, Orlando, USA; 3 Internal Medicine, Abington Hospital - Jefferson Health, Abington, USA; 4 Internal Medicine, University of Missouri-Kansas City School of Medicine, Kansas City, USA; 5 Gastroenterology, AdventHealth, Orlando, USA

**Keywords:** gastroparesis, refractory gastroparesis, postsurgical gastroparesis, botulinum toxin therapy, endoscopic botox injection

## Abstract

Gastroparesis is a complex dysmotility disorder characterized by chronic dyspepsia and delayed gastric emptying in the absence of mechanical obstruction. Postsurgical gastroparesis is the third most common cause and accounts for 13% of total cases. Studies have shown that catheter ablation procedures for atrial fibrillation can rarely result in gastroparesis, secondary to damage to the vagus nerve. Once the diagnosis is confirmed, treatment options include: dietary management, prokinetic drugs, and new invasive treatments. Botulinum toxin injection is an emerging pyloric intervention, increasingly used in the management of gastroparesis refractory to pharmacological therapy. It is given as an injection into the pyloric sphincter, with the help of an endoscope. Botulinum toxin acts by inhibiting smooth muscle contraction through a decreased response to acetylcholine. Here we report a case of postsurgical gastroparesis that responded well to botulinum toxin therapy.

## Introduction

Gastroparesis is a complex dysmotility disorder characterized by chronic dyspepsia and delayed gastric emptying in the absence of obstruction due to mechanical reasons [1]. Symptoms comprise of postprandial fullness and early satiety associated with nausea, vomiting, bloating, and epigastric pain. Etiology includes: idiopathic (no primary cause), diabetes, postsurgical, medication induced, neuromuscular causes, and collagen vascular diseases like scleroderma [2].

Postsurgical gastroparesis is the third most common cause and accounts for 13% of total cases [3]. Studies show that catheter ablation procedures for atrial fibrillation can rarely result in gastroparesis, secondary to damage to the vagus nerve [4].

Botulinum toxin injection is an emerging pyloric intervention, increasingly used in the management of gastroparesis refractory to pharmacological therapy [2]. It is given as an injection into the pyloric sphincter, with the help of an endoscope. Botulinum toxin acts by inhibiting smooth muscle contraction through a decreased response to acetylcholine [2]. Here we report a case of postsurgical gastroparesis that responded well to botulinum toxin therapy.

## Case presentation

**Figure 1:**
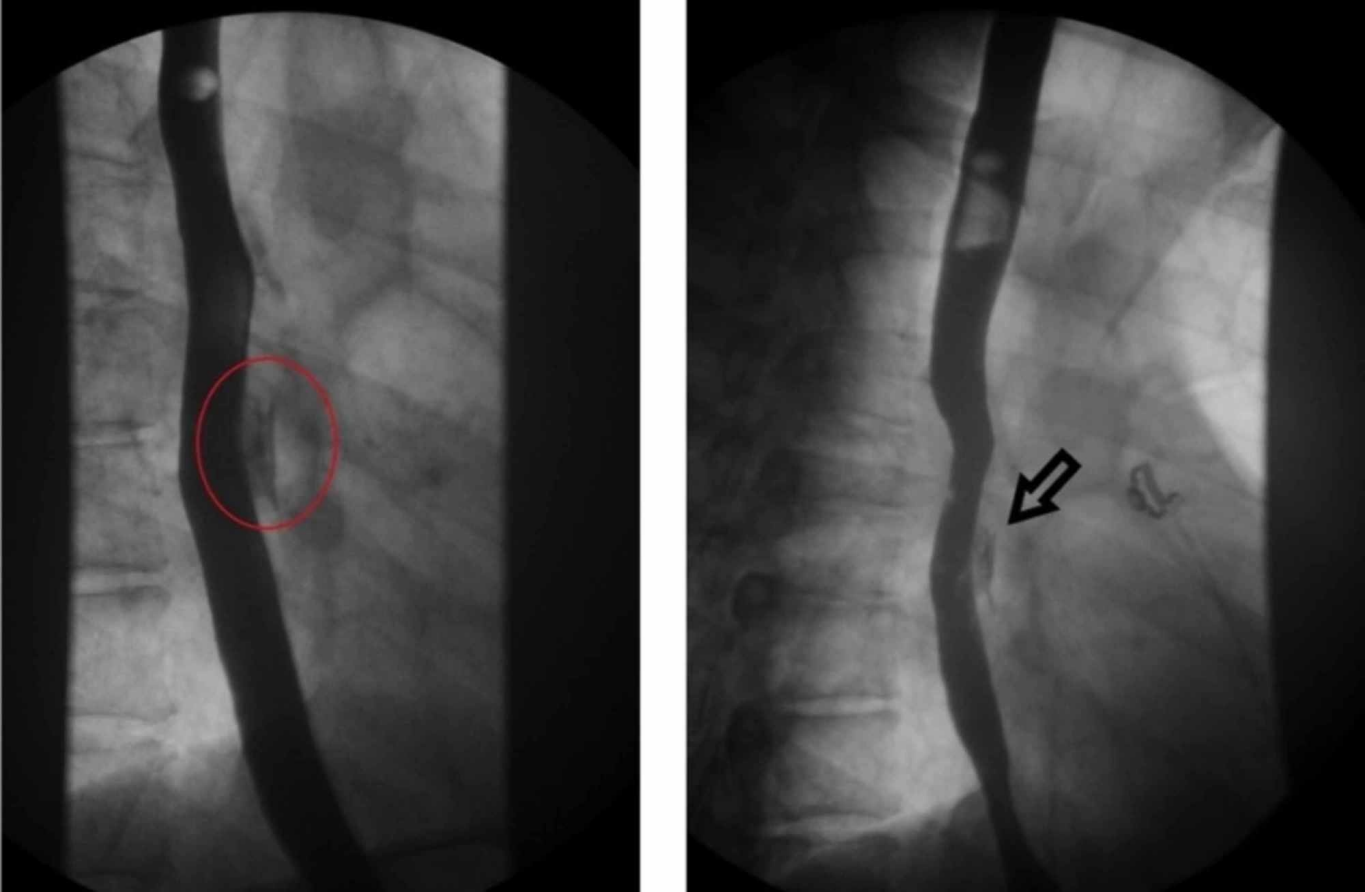
Upper GI series demonstrating esophageal tear with extravasation of contrast - red circle area (left image) and arrow (right image) GI: Gastrointestinal

**Figure 2:**
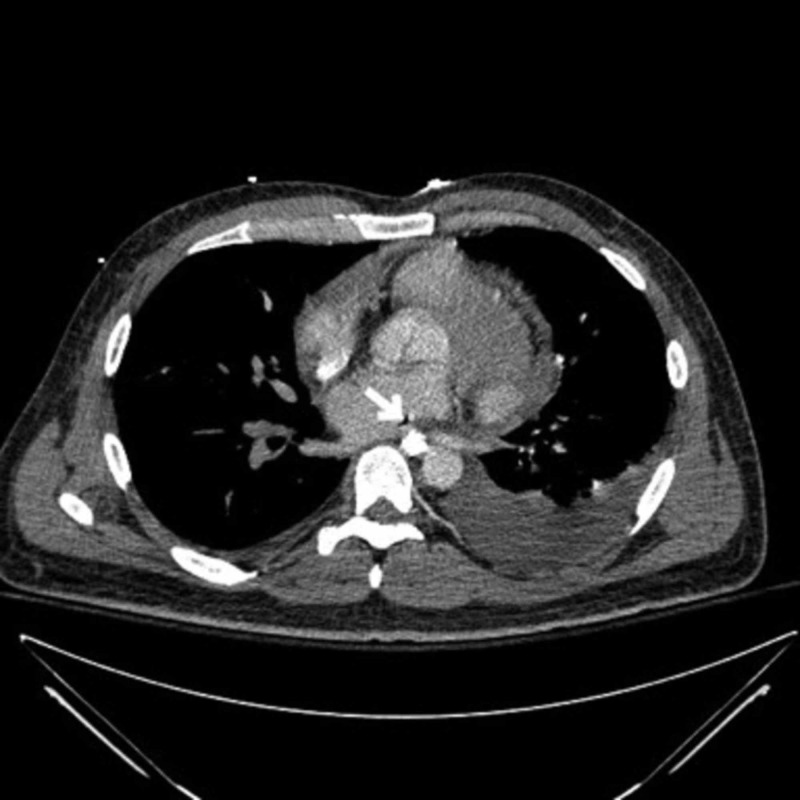
Chest CT (pulmonary veins) showing atrio-esophageal fistula (white arrow) CT: Computerized tomography

A 43-year-old gentleman with a past medical history of atrial fibrillation and status/post cardiac ablation four days prior, presented to the emergency department with complaints of retrosternal chest pain and odynophagia [5]. Initial workup ruled out myocardial infarction and the patient was diagnosed with linear deep mid-esophageal tear (Figure 1). Later, the esophageal tear was closed with six hemostatic clips and the patient was discharged on total parenteral nutrition. Two weeks later, he presented to the emergency department again for dyspnea and chest discomfort. On exam, the patient had loud pericardial friction rub, and stat echocardiogram showed pericardial effusion with early signs of cardiac tamponade [5]. The patient was treated with emergent pericardiocentesis but soon after the procedure, he became febrile and chest CT (pulmonary veins) revealed atrio-esophageal fistula (Figure 2), pericardial effusion, and left-sided pleural effusion with empyema [5]. The patient was managed with thoracotomy, decortication of empyema, and repair of inferior pulmonary vein/atrial margin, esophageal repair, gastro-jejunal (G/J) tube placements, and omental transfer. The patient tolerated the procedures well but soon developed gastroparesis [5]. After other causes of gastroparesis were excluded, the patient’s gastroparesis was supposed to be due to vagal nerve injury postoperatively. Subsequently, the patient received intrapyloric sphincter botulinum toxin injection. He responded well to the therapy and his gastroparesis improved by 60% [5].

## Discussion

Gastroparesis is a challenging disorder of delayed gastric emptying that impairs the patient’s quality of life. Based on a community study, the age-adjusted prevalence of gastroparesis for men and women per 100,000 was 9.6 and 37.8, respectively [6]. Patients often present with postprandial fullness, nausea, vomiting, early satiety, and epigastric pain. Etiology includes idiopathic (post-viral and post-bacterial), autonomic neuropathy secondary to diabetes, Parkinson's disease, scleroderma and medications [2]. Three most common causes are: diabetes (29%), postsurgical (13%), and idiopathic (36%) [3,5].

Postsurgical gastroparesis is the third most common cause of gastroparesis. It occurs following vagus nerve injury or vagotomy [5]. Catheter ablation is a routine procedure for the treatment of atrial fibrillation. One of the rare complications of the procedure is gastroparesis. The prevalence is reported at 10% and 6% respectively for radiofrequency ablation and cryoablation procedure [4]. Periesophageal vagal nerve injury during the procedure is the probable mechanism; the vagus nerve runs close to the left atrium and supplies the stomach and pyloric sphincter. Patients usually manifest with symptoms of delayed gastric emptying within a few days following the procedure.

The diagnostic criteria of gastroparesis are a combination of symptoms of gastroparesis, absence of mechanical gastric outlet obstruction, and an objective delay in gastric emptying. Objective confirmation of delayed gastric emptying is crucial before making a diagnosis of gastroparesis since other conditions present with similar symptomatology such as Helicobacter pylori infection, peptic ulcer disease, gastroesophageal reflux disease, and functional dyspepsia [1]. A delay in four-hour scintigraphic gastric emptying of solids confirms the diagnosis. Besides, screening for diabetes mellitus, thyroid dysfunction, neurological disease, and autoimmune disorders should be performed in all patients through standard endoscopy, lab studies and CT of head and abdomen [1,5].

Once the diagnosis is confirmed, treatment options include dietary management (low fat, low fiber diet), fluid and electrolyte replacement, prokinetic drugs like metoclopramide and erythromycin, and new invasive treatments [5]. Invasive options consist of novel and emerging therapies such as pyloric botulinum toxin injection, venting gastrostomy, feeding jejunostomy, pyloroplasty and gastric electrical stimulation [7]. Even though prokinetic agents are the mainstay of treatment in gastroparesis after diet failure, the side effects and lack of effectiveness limit their long-term use [7]. Botulinum toxin injection is an emerging and very safe pyloric intervention effective in the treatment of refractory gastroparesis that does not respond to medications.

Botulinum toxin is a bacterial neurotoxin with strong paralytic effects on the skeletal muscle. The effects of botulinum on smooth muscle were tested in two in vivo studies. The toxin was able to decrease the lower esophageal sphincter pressure by 60% and also the sphincter of Oddi pressure by 50% when compared to placebo [8,9]. Later, studies on pyloric muscle strips demonstrated the potential of botulinum toxin in decreasing contractions induced by acetylcholine and substance P [10]. In addition, the effects of botulinum toxin-A (BT-A) are dose-dependent. At low doses, it causes inhibition of acetylcholine release from cholinergic nerve endings via calcium channels, and direct inhibition of smooth muscle contraction was observed at higher doses [10]. Based on the existing data, botulinum toxin is a safe drug with very few adverse reactions. The medication is injected endoscopically, into the four quadrants of the pylorus, with the help of a sclerotherapy needle. A total dose of 100-200 U can be injected in each session [11]. In patients with gastroparesis, botulinum toxin acts by relaxing the hypertonic smooth muscle in the stomach, through a decreased response to acetylcholine [12].

The existing literature regarding the effectiveness of botulinum toxin injections is limited to open-label studies and randomized controlled trials in small patient populations. In 2002, Ezzeddine et al. published the first report on the effect of botulinum toxin in gastroparesis patients [13]. They conducted an open-label trial on six male patients with diabetes (mean age = 62 years), who were diagnosed with gastroparesis on solid-phase gastric emptying study. The patients received 100 U of botulinum toxin and the solid phase gastric emptying study was repeated at 48 h and six weeks after the treatment. Gastric emptying rate, which was 27.8% before the procedure, improved to 44.4% at 48 h and 49% at six weeks [13]. However, the study was limited in terms of fewer study population and lack of control group. An open-label study was done retrospectively in an institute with 179 patients where more than half of the patients experienced positive clinical response from the treatment. 51.4% of the patients reported decreased symptoms of gastroparesis one to four months after intrapyloric botulinum toxin therapy [2]. The benefit was greater in females, age < 50 years and with a 200-unit compared to a 100-unit dose, and idiopathic gastroparesis; furthermore, a second dose of injection produced a positive clinical response in 73.4% of the patients [2].

The success of the open-label studies motivated the researchers to conduct randomized controlled trials. Friedenberg et al. conducted a study on 32 patients who were randomly assigned to either 200 U of botulinum toxin or placebo [14]. The primary endpoint of the study was a nine-point decrease in symptom score at the end of one month. While only six patients in the botulinum toxin group showed clinical improvement, it bumped to nine patients in the placebo group. The gastric emptying rate improved in the botulinum toxin group but the results were not statistically significant. A recent meta-analysis of 15 studies showed a beneficial effect of botulinum toxin-A treatment for gastroparesis [15]. Almost 51% of people noticed significant clinical improvement and the benefit was greater in females, younger patients (age < 50 years), idiopathic gastroparesis and those who received a higher dose of medication. In addition, 87 patients received repeat injections (150 or 200 U) and the benefit was similar to the first dose effect [15].

## Conclusions

In conclusion, gastroparesis is a complex motility disorder where a mechanical cause of obstruction is not identified. People who are young (<50 years age), female, non-diabetic and those refractory to medical therapy are more likely to benefit from botulinum toxin therapy. Given the safety of the drug, botulinum toxin treatment can be a good option for the patients when they fail other types of pharmacological therapy.
